# Modulation of Cell Cycle Profile by *Chlorella vulgaris* Prevents Replicative Senescence of Human Diploid Fibroblasts

**DOI:** 10.1155/2013/780504

**Published:** 2013-03-12

**Authors:** Tayyebeh Saberbaghi, Firouz Abbasian, Yasmin Anum Mohd Yusof, Suzana Makpol

**Affiliations:** ^1^Department of Biochemistry, Faculty of Medicine, Universiti Kebangsaan Malaysia, Jalan Raja Muda Abdul Aziz, 50300 Kuala Lumpur, Malaysia; ^2^Department of Microbiology, Science and Research Branch, Islamic Azad University, P.O. Box 14155/4933. 3, Tehran, Iran

## Abstract

In this study, the effects of *Chlorella vulgaris* (CV) on replicative senescence of human diploid fibroblasts (HDFs) were investigated. Hot water extract of CV was used to treat HDFs at passages 6, 15, and 30 which represent young, presenescence, and senescence ages, respectively. The level of DNA damage was determined by comet assay while apoptosis and cell cycle profile were determined using FACSCalibur flow cytometer. Our results showed direct correlation between increased levels of damaged DNA and apoptosis with senescence in untreated HDFs (*P* < 0.05). Cell cycle profile showed increased population of untreated senescent cells that enter G0/G1 phase while the cell population in S phase decreased significantly (*P* < 0.05). Treatment with CV however caused a significant reduction in the level of damaged DNA and apoptosis in all age groups of HDFs (*P* < 0.05). Cell cycle analysis showed that treatment with CV increased significantly the percentage of senescent HDFs in S phase and G2/M phases but decreased the population of cells in G0/G1 phase (*P* < 0.05). In conclusion, hot water extract of *Chlorella vulgaris* effectively decreased the biomarkers of ageing, indicating its potential as an antiageing compound.

## 1. Introduction

Regardless of the persons' official age, senescence is associated with appearance of age-related phenotypes, decline in protein homeostasis, and accumulation of DNA damage which alters individual lifespan. The theories of ageing can be classified into two broad categories, namely, the genetic and stochastic theories [1]. The genetic theories postulated that ageing process is under the control of the same genes responsible for running the life cycle program [2]. However, the stochastic theories rely on the accumulation of cell damages and their inefficient repair as the main reason of ageing [3]. In other words, we can do nothing with ageing based on the genetic theories, but it is reversible based on the stochastic theories by improving the cellular defense system or decreasing cell damages.

Based on the free radical theory of ageing, accumulation of free radicals over times leads to cellular damages and progressive deterioration of cells functions, tissues, and organ systems [4]. Due to their instability, the free radicals will attack the neighboring molecules to balance their unpaired electrons. Free radicals can be produced intrinsically through normal metabolic processes or extrinsically from exogenous agents [5, 6]. The mitochondria, for instance, are major contributors of reactive oxygen species (ROS) in the cells [4], and any dysfunction in this organelle can result in energy shortage and other cellular deficiency that finally give rise to age-related disorders such as muscular and neurological degeneration, heart failure, strokes, and other degenerative diseases and finally death.

Therefore, introducing natural or synthetic compounds with antioxidant properties may be beneficial to protect cells or tissues against oxidative damage which may result in age-related diseases as well as promoting longevity. Previous study showed that antioxidant activity of resveratrol in grapes and tocotrienol in palm oil extends lifespan [7–10]. 

*Chlorella vulgaris *(CV) is a unicellular green microalgae which has the ability to stimulate immune system [11], improve collagen synthesis [12], and prevent apoptosis [13]. Furthermore, since CV exhibits high antioxidant activity [14], its radical scavenging activity has been manipulated to promote its anticancer and antiatherogenic properties [15]. In this study, the antiageing properties of CV were investigated by elucidating its effects on senescence biomarkers: DNA damage, cell cycle profile, and apoptosis.

## 2. Material and Methods

### 2.1. *Chlorella vulgaris* Hot Water Extracts Preparation

A substrain of green unicellular microalgae *Chlorella vulgaris* Beijerinck grown in Bold's Basal Media was subjected to hot water extraction as previously described [16]. Briefly, dried *C. vulgaris* cells were suspended in distilled water at a concentration of 10% w/v, boiled at 100°C for 20 min, and then centrifuged at 10,000 rpm for 20 min. The supernatant was subsequently lyophilized to obtain *C. vulgaris* extracts (CVE) which was then added to cell culture growth medium as supplementation.

### 2.2. Cell Culture and Treatment Protocol

Human diploid fibroblasts (HDFs) were obtained from the foreskin of 8- to 12-year-old boys who underwent circumcision. Written informed consent was obtained from parents of all subjects. The HDFs were cultured till different passages (P6, P15 and P30), representing different age groups (young, pre-senescent, and senescent, resp.). Each age group was divided into two subgroups, treated with CV and untreated control. Young, presenescent, and senescent HDFs were treated with 400 *μ*g/mL, 200 *μ*g/mL and 100 *μ*g/mL of *Chlorella vulgaris*, respectively for 24 h before determination of aging biomarkers which include morphological analysis, senescence-associated *β*-galactosidase (SA-B-gal) expression, DNA damage, apoptosis, and cell cycle profile. The different dosages used for CV treatment were obtained from viability assay. This research has been approved by the Ethics Committee of the Universiti Kebangsaan Malaysia (approval project code: FF-028-2012). 

### 2.3. Senescence-Associated Beta-Galactosidase (SA-*β*-gal) Staining

Expression of SA-*β*-gal was determined based on the method suggested by Dimri et al. [17] with minor modification. HDFs (1 × 10^5^) were inoculated in 6-well plates and treated with fixation buffer containing 0.2% glutaraldehyde and 2% formaldehyde. After incubation for 6-7 min at room temperature, the cells were washed with PBS (three times) and incubated for 4 h at 37°C (without CO_2_) in buffer containing 1 mg/mL 3-indolyl-beta-D-galactopyranoside, 40 mM citric acid/phosphate (pH 6.0), 2 mM MgCl_2_, 5 mM K_3_FeCN_6_, and 5 mM NaCl. Blue staining was visible after incubation, and the percentage of blue cells observed in 100 cells under a light microscope was calculated. 

### 2.4. Determination of DNA Damage

DNA damage was determined by single cell gel electrophoresis (SCGE) assay also known as the comet assay according to the method previously described by Singh et al. [18] with slight modifications. Briefly, 1 × 10^4^ cells in 5 *μ*L medium were suspended in 70 *μ*L warm 0.6% low-melting point (LMA) agarose (Boehringer Mannheim, Germany) (DNAse-free, RNAse-free) and was quickly pipetted onto a prepared frosted slide coated with 80 *μ*L of 0.6% normal-melting (NMA) agarose. Slides with cell suspension were covered with glass coverslips and subsequently placed in a Coplin jar containing chilled lysis solution (10% DMSO, 1% Triton X in alkaline lysis buffer: 2.5 M NaCl, 10 mM Tris, 100 mM Na_2_EDTA, pH 10) for 1 h to completely digest cellular proteins. Next, the slides were removed from lysis solution and placed in a horizontal gel electrophoresis unit filled with cold electrophoresis buffer (10 mM NaOH and 200 mM Na_2_EDTA at pH 13.2). Slides were allowed to sit in the alkaline buffer for 20 min to allow unwinding of DNA strands and expression of alkali-labile damage. Electrophoresis was performed for 20 min at 300 mA and 25 V. Following dropwise neutralization (Tris-HCl, pH 7.5) for 5 min, cells were stained by applying 30 *μ*L 1X ethidium bromide. The slides were examined and the tail length was measured with a fluorescence microscope (Carl Zeiss, Germany). The DNA migration of 100 randomly selected cells was examined for each sample. A total damage score was determined by multiplying the number of cells assigned to each grade of damage by the numeric value of the grade according to methods described by Heaton et al. [19]. Total DNA damage score was calculated as follows.

Total DNA damage = [(0 × *n*_0_) + (1 × *n*_1_) + (2 × *n*_2_) + (3 × *n*_3_) + (4 × *n*_4_)] where *n*_0_ = cells with Score 0, *n*_1_ = cells with Score 1, *n*_2_ = cells with Score 2, *n*_3_ = cells with Score 3, and *n*_4_ = cells with Score 4.

### 2.5. Apoptosis Determination by Annexin V-FITC

Externalization of phosphatidylserine (PS) at the cell surface of HDFs as an early apoptotic event was assessed by Annexin V-FITC Apoptosis Detection Kit II (BD Pharmingen, San Diego, CA, USA) according to the manufacturer's instructions. Cells were washed twice with cold PBS and resuspended in 1X binding buffer to prepare 1 × 10^6^ cells/mL concentration. Then 5 *μ*L of Annexin V-FITC and 5 *μ*L of PI staining solution were added into a 5 mL culture tube containing 100 *μ*L of cells suspension, followed by incubation for 15 min in the dark at room temperature (25°C). Finally, cells were suspended in 400 *μ*L 1X binding buffer and analyzed within 1 h by FACSCalibur flow cytometer (Becton Dickinson, San Jose, CA, USA).

### 2.6. Determination of Cell Cycle Profile

HDFs were harvested at desired time points after trypsinization and were rinsed 3 times with buffer solution with adjusted concentration 1 × 10^6^ cells/mL and prepared using CycleTEST PLUS DNA Reagent Kit (Becton Dickinson, USA) according to the manufacturer's instruction. Cell cycle status was analyzed by flow cytometer using propidium iodide (PI) as a specific fluorescent dye probe. The PI fluorescence intensity of 10,000 cells was measured for each sample using a Becton-Dickinson FACSCalibur flow cytometer.

### 2.7. Statistical Analysis

Each experiment was carried out in duplicates from three independent cultures. Data were reported as means ± SD. Comparison between groups was made by Student's *t*-test (two-tailed). *P* < 0.05 was considered statistically significant

## 3. Results

### 3.1. Senescence-Associated Beta-Galactosidase (SA-*β*-gal) Expression

**Figure 1:**
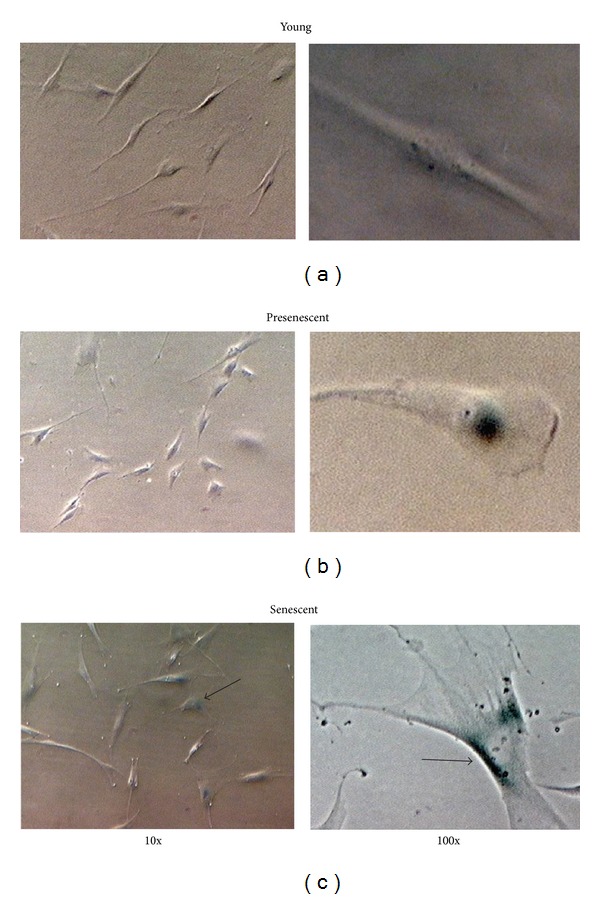
Morphological changes in HDFs in culture. Senescence-associated *β*-galactosidase (SA *β*-gal) staining. Positive blue stains of SA *β*-gal appeared in senescent HDFs as indicated by arrows. Micrographs are shown at 10x and 100x magnifications.

**Figure 2:**
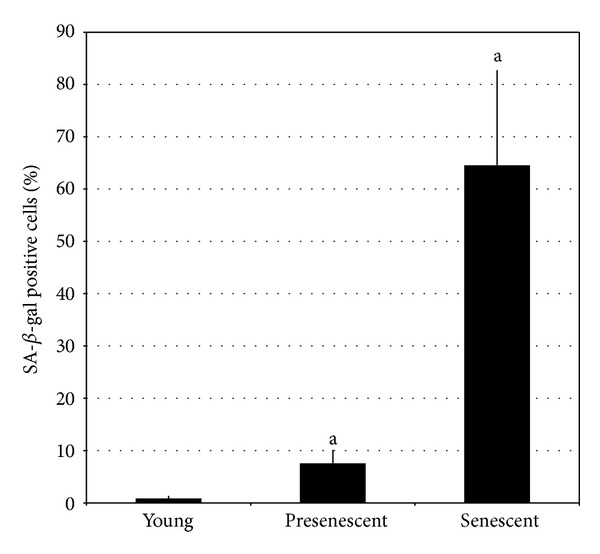
Quantitative analysis of positive SA *β*-gal stained cells. Data are expressed as means ± SD, *n* = 6. ^a^Denotes *P* < 0.05 compared to young HDFs.

Young HDFs display spindle-shaped morphology while senescent fibroblasts were larger and flattened in shape containing cytoplasmic inclusions (Figure 1). Senescent HDFs at passage 30 contained numerous blue stain granules compared to HDFs at lower passages (young and presenescent HDFs). Quantitative analysis showed a significant increase in SA-*β*-gal in pre-senescent and senescent HDFs as compared to young cells (*P* < 0.05) (Figure 2). 

### 3.2. Determination of DNA Damage

**Figure 3:**
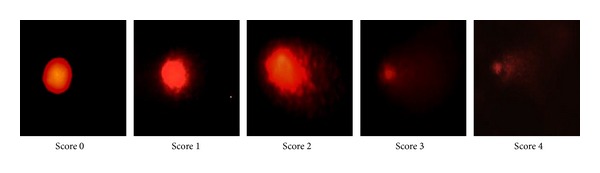
DNA damage. The cell nuclei were classified into five categories: (Score 0), undamaged (nuclei without comet tail); (Score 1), low damaged (nuclei with comet tails up to two- fold longer than nucleus diameter); (Score 2), damaged (nuclei with comet tail two- to threefold longer than nucleus diameter); (Score 3), highly damaged (nuclei with comet tails threefold longer than nucleus diameter); and (Score 4), severely damaged (cell nuclei were almost not visible with long and dispersed comet tails).

**Figure 4:**
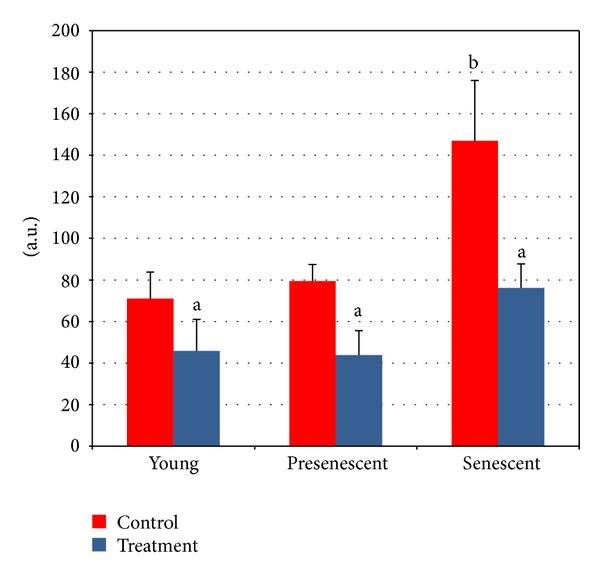
Protective effects of CV against DNA damage in young, presenescent, and senescent HDFs. ^a^Denotes *P* < 0.05 compared to untreated HDFs, ^b^ *P* < 0.05 compared to untreated young HDFs. Data are expressed as means ± SD, *n* = 6.

 The cell nuclei were classified into five categories: (0), undamaged (nuclei without Comet tail); (1), low damaged (nuclei with Comet tails up to two fold longer than nucleus diameter); (2), damaged (nuclei with Comet tail two- to threefold longer than nucleus diameter); (3), highly damaged (nuclei with Comet tails threefold longer than nucleus diameter); and (4), severely damaged (cell nuclei were almost not visible with long and dispersed Comet tails) (Figure 3). Senescent cells showed a significant increase in damaged DNA as compared to young and pre-senescent cells (*P* < 0.05). Treatment with CV, however, significantly decreased the damaged DNA in all age groups of HDFs (*P* < 0.05) (Figure 4).

### 3.3. Apoptotic Changes Detected by Annexin V-FITC

**Figure 5:**
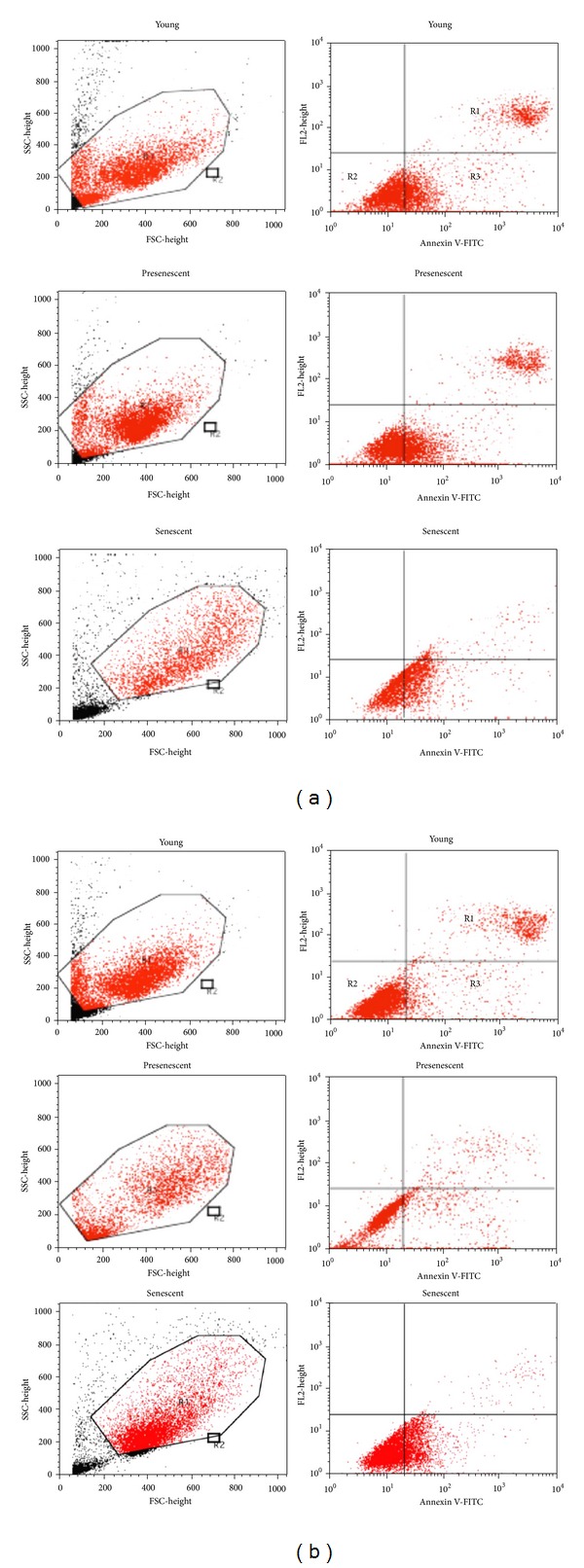
Contour diagram of FITC-Annexin V/PI double staining by flow cytometry. The three quadrants represent different cells conditions the upper right quadrant (R1), nonviable, late apoptotic, and necrotic cells (FITC^+^/PI^+^); lower left quadrant (R2), viable cells (FITC^−^/PI^−^) and lower right quadrant (R3), early apoptotic cells (FITC^+^/PI^−^). (a) Untreated control HDFs. (b) CV-treated HDFs.

**Figure 6:**
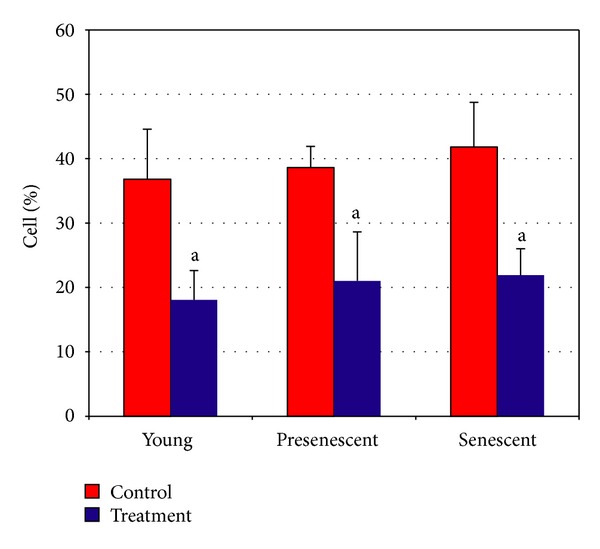
Percentage of cells at early apoptotic stage demonstrated by FITC^+^/PI^−^. ^a^Denotes *P* < 0.05 compared to untreated HDFs. Data are expressed as means ± SD, *n* = 6.

Figure 5 shows the contour diagram of FITC-Annexin V/PI double staining by flow cytometry analysis. The three quadrants represent different cells conditions; the upper right quadrant (R1) indicates nonviable, late apoptotic, and necrotic cells (FITC^+^/PI^+^), lower left quadrant (R2) indicates viable cells (FITC^−^/PI^−^) and lower right quadrant (R3) indicates early apoptotic cells (FITC^+^/PI^−^), which is demonstrated by Annexin V binding and cytoplasmic membrane integrity. The percentage of cells at early apoptotic stage in all age groups was significantly decreased in HDFs treated with CV as compared to untreated control (*P* < 0.05) (Figure 6). 

### 3.4. Cell Cycle Profile Analysis

**Figure 7:**
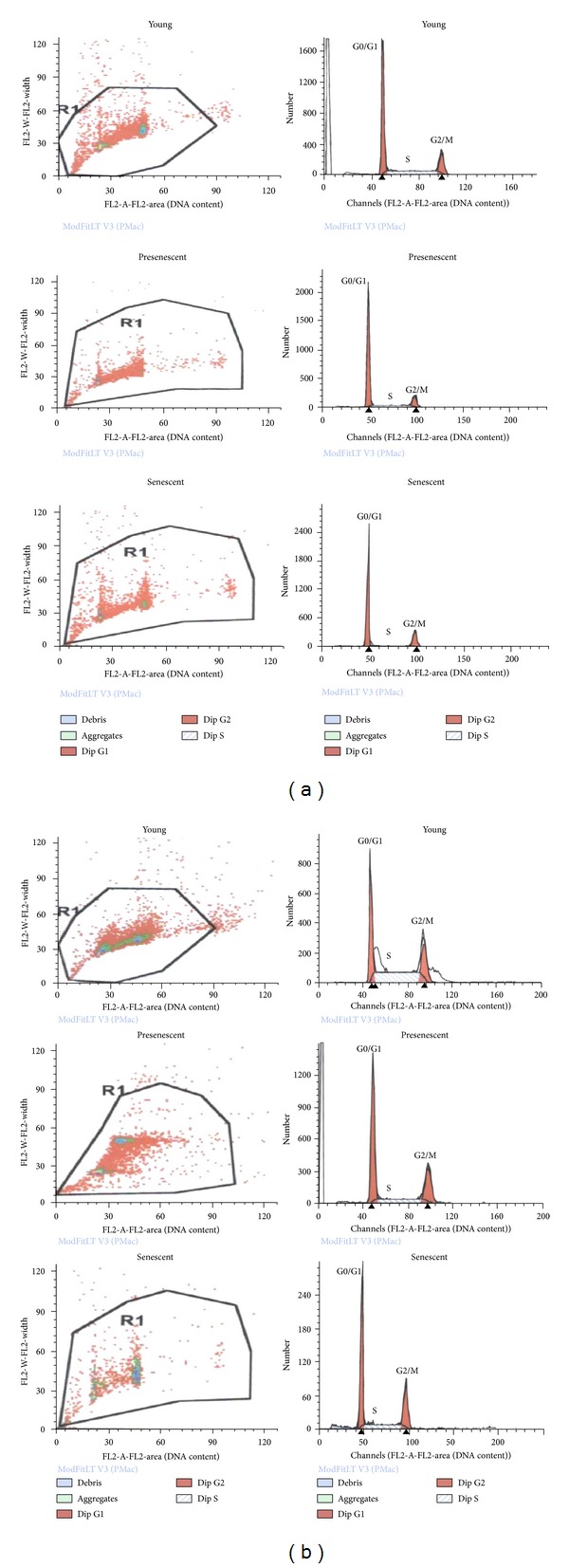
Flow cytometry analysis of cell cycle progression in young, presenescent, and senescent HDFs. (a) Untreated HDFs. (b) CV-treated HDFs.

**Figure 8:**
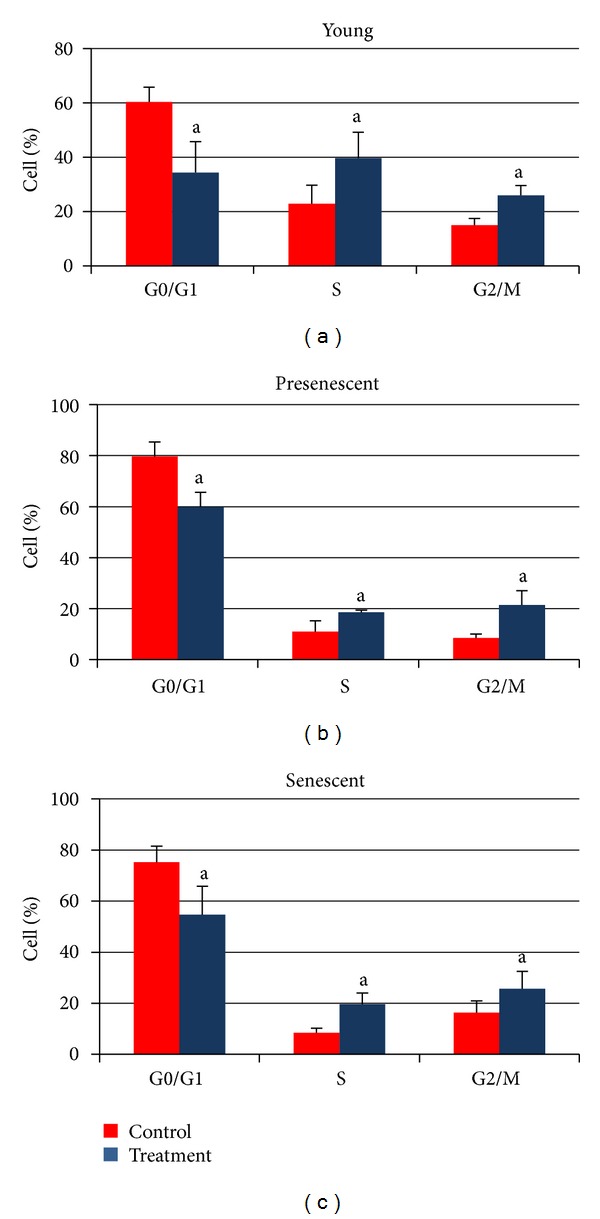
Quantitative analysis of cell cycle progression in young, presenescent, and senescent HDFs with and without CV treatment. ^a^Denotes *P* < 0.05 compared to untreated HDFs. Comparison was done between HDFs in the same phase of cell cycle. Data are expressed as means ± SD, *n* = 6.

Analysis on cell cycle profile showed that HDFs population in G0/G1 phase was significantly increased while S phase and G2/M phase cells decreased in senescent cells as compared to young HDFs (Figures 7 and 8) (*P* < 0.05). Treatment with CV significantly increased the number of cells in S phase and G2/M phase (*P* < 0.05) in all age groups of HDFs. In contrast, population of cells in G0/G1 phase decreased significantly (*P* < 0.05) after CV treatment in all age groups (Figure 8).

## 4. Discussion

For the past few decades, researchers have tried to find a way to postpone ageing and extend lifespan. Findings from ageing research provide promising data to prolong maximum lifespan of human being [20]. Normal metabolic processes which involved respiratory reactions in electron transport chain in the mitochondria produce reactive oxygen species (ROS) such as O_2_^−^, H_2_O_2_, ROO^−^, OH^•^, and O_3_^−^. These free radicals are hazardous to cells and can initiate senescence pathway [21, 22]. Although eukaryotic cells are able to remove the free radicals, their ability is limited as they cannot cope with the deleterious effects of accumulated free radicals during life [23, 24]. Accumulation of free radicals causes damage to DNA due to increase in mutation rate and cells' inability to repair the damaged DNA [25]. Damaged DNA will further activate tumor suppressor genes such as p53 and Rb and halt cell proliferation, leading to senescence [23, 25, 26]. Therefore, reduction of oxidative stress by an antiageing compound is considered as an effective antiageing intervention.

Several antiageing compounds and natural products have been identified which reduced the oxidative damage of mitochondrial DNA and decreased mitochondrial reactive oxygen species production [27, 28]. For instance, vitamin E has been proven to have antiageing properties. Tocotrienol, a type of vitamin E, was reported to prevent telomere shortening [29] and decreased oxidative damage in ageing model [27]. Another natural product with antiageing properties is garlic which acts as an efficient anti-inflammatory factor and an antioxidant agent that can remove reactive oxygen species and peroxyl radicals [30, 31]. Similar effects have been shown by Curcumin derived from turmeric [32, 33]. Therefore introducing antioxidant compounds will improve physiological functions of cells and delay the onset of age-related phenomenon in the cells [34]. 

*Chlorella vulgaris* (CV) is a spherical (2–10 *μ*m) and nonflagellated green algae belonging to the phylum Chlorophyta. It contains proteins, fibers, minerals, vitamins, and chlorophylls and is considered as a rich source of antioxidant such as *α*- and *β*-carotene, lutein, ascorbic acid, and vitamin E [35–37]. CV is well recognized as a biological modifier that can modulate immune responses against viruses, bacteria, and tumors by increasing CD^4−^CD^8+^ T cells, granulocytes, and macrophage [11, 38, 39]. Because of its nutritional values, CV has been used as a supplement in many countries such as Japan, Taiwan, and the USA [11, 38, 40]. 

Therefore CV with its antioxidant properties may contribute in postponing ageing by decreasing oxidative stress in cells. In this study, the effects of CV on a few ageing biomarkers including DNA damage, apoptosis, and cell cycle arrest were evaluated. Primary human diploid fibroblast cells derived from foreskin were cultured until passage 30 which represents cellular senescence. Senescent cells were confirmed by the presence of SA-*β*-gal which forms aggregates in autophagic lysosomal vacuoles when cells reached senescence [41–43]. The findings of this study showed that 64.6% of the cells in passage 30 were stained for SA-*β*-gal indicating senescent cells. It has been reported that increased autophagic vacuoles and *β*-galactosidase activity during cell ageing are associated with increase in lysosomal mass and accumulation of lipofuscin [43, 44].

 Since DNA damage is one of the critical factors that trigger ageing, we also investigated the effects of CV on DNA damage. Our results showed increased damaged DNA with cellular ageing and CV could significantly reduce DNA damage in all age groups of HDFs. This could be due to its antioxidant properties which quenched the reactive ROS and free radicals from attacking the DNA. Thus CV conserves the DNA from destruction and can effectively delay cellular ageing.

Our results showed an extensive decline in the percentage of senescent HDFs that enter into S phase while the percentage of cells in G0/G1 phase was increased with senescence. CV treatment to HDFs increased the percentage of cells that enter into S and G2/M phases and at the same time decreased the percentage of cells that enter into G1 phase. The direct relationship between decreased DNA damage and increased cell replication suggests improvement in cell division. This could be due to a more efficient repair of the DNA or prevention of DNA damage in cells treated with CV.

In addition, since programmed cell death or apoptosis is the outcome of DNA damage and normally occurs in more than 10% of all living cells [21], the effect of CV on this parameter was also investigated. Our findings showed that CV could effectively decrease apoptosis in all stages of ageing (young, pre-senescence, and senescence). Therefore in senescence, the level of damaged DNA increases, which leads to apoptosis. Subsequently the cells can be directed to two different fates: either they undergo senescence or may return to a replicative life cycle. Our data on cell cycle profile suggested that CV returns the HDF cells back to the replicative state and thus prevents cellular ageing.

## 5. Conclusion

Hot water extract of *Chlorella vulgaris *(CV) is able to delay cellular ageing by decreasing the level of DNA damage and apoptosis as well as promote cell cycle progression. Therefore, *Chlorella vulgaris* extracts are a potent antiageing compound; however, further studies are needed before it can be introduced as an effective antiageing supplement.
